# Instrumental and Sensory Analyses of Salami from Autochthonous and Conventional Pig Breeds

**DOI:** 10.3390/foods11142060

**Published:** 2022-07-12

**Authors:** Enrico Valli, Federica Tesini, Matilde Tura, Francesca Soglia, Massimiliano Petracci, Alessandra Bendini, Tullia Gallina Toschi

**Affiliations:** 1 CIRI-Agrifood (Interdepartmental Centre of Industrial Agrofood Research), Alma Mater Studiorum—Università di Bologna, 47521 Cesena, Italy; enrico.valli4@unibo.it (E.V.); tesini.federica@gmail.com (F.T.); francesca.soglia2@unibo.it (F.S.); m.petracci@unibo.it (M.P.); alessandra.bendini@unibo.it (A.B.); tullia.gallinatoschi@unibo.it (T.G.T.); 2 Department of Agricultural and Food Sciences, Alma Mater Studiorum—Università di Bologna, 47521 Cesena, Italy

**Keywords:** Mora Romagnola salami, sensory analysis, textural properties, typical product, volatile compounds, electronic eye

## Abstract

Typical salami produced from Mora Romagnola (MR), an autochthonous pig breed extensively farmed within a geographically confined Italian area, are food products of commercial interest. This investigation aimed to highlight elements for the recognition and authentication of such typical salami. Five MR salami were analyzed using a sensory and instrumental approach, and the results were compared with those from eight salami made from a conventional pig breed. The sensory profiles were defined through attributes such as seasoning, pepper, garlic, fermented etc.; no differences in the products ascribable to the pig breed were highlighted. By analyzing volatile compounds, 33 molecules were detected; significant differences were found among samples, probably related to processing methods. Color differences between MR and conventional salami were identified by an electronic eye. According to instrumental texture analysis, large variability among the MR samples was detected, probably related to different types of salami (recipe, casing, size, ripening). Correlations were found among the sensory results, volatile compounds, and textural properties of the samples. Most differences do not appear to be specifically related to MR breed; only red color intensity evaluated by an electronic eye showed a correlation with breed, i.e., MR salami. This study highlights the large variability among the salami analyzed.

## 1. Introduction

The concept of local food, traditionally related to geographical origin, constitutes an important driver for consumer demand of typical products [1]. Moreover, aspects such as the history of preparation of a typical product, together with specific ingredients, represent an important part of quality perception in food [2,3]. Traditional products are enriched by added cultural and identity values, as they contribute to the maintenance and development of the rural areas of their origins [4].

The word “salami” is used to define a specific type of dry fermented sausage that is air-dried or smoked and ripened for long time. Salami consumption, in Italy, is a perfect example of something that was historically considered to be a traditional national food that tends to differ from one region to another [5,6,7]. In the past few years, there has been an increasing interest in the development of meat products manufactured with raw meat obtained from pigs of the same local or autochthonous breed [8].

Nowadays, in Mediterranean countries, the production of traditional dry-cured meat products, including dry-fermented sausages, which was previously based on local breeds, largely depends on commercial pig breeds and hybrids farmed under intensive production systems [9]. Indeed, even in Italy, the current pork-meat supply used to manufacture typical meat products relies on very few pig breeds [10], although it is well established that different genotypes (i.e., genetic diversity within species) can provide significant nutritional variation and counteract the loss of nutritional biodiversity in modern societies [11]. The lipid content of pork meat varies based on genotype, with different breeds containing different individual fatty acids, minerals, and vitamins [10,12,13]. In this context, only six (i.e., Apulo-Calabrese, Casertana, Cinta Senese, Mora Romagnola, Nero Siciliano, and Sarda) of the several native pigs identified by the Food and Agriculture Organization of the United Nations (FAO) are currently used for local food production [13,14]. Mora Romagnola is an autochthonous pig breed farmed in the Emilia-Romagna region under extensive or semi-extensive systems and used to produce traditional products (i.e., dried hams and salami) [15,16]. Recently, the Mora Romagnola Consortium has been founded to promote the visibility of these typical products and protect them from potential fraud; however, there is a lack of information concerning their quality and, in particular, sensory traits and linkages with a specific geographic origin (i.e., Protected Designation of Origin PDO and Protected Geographical Indication PGI) [15]. 

The sensory characteristics of dry-fermented salami derive from a large number of biochemical and physicochemical transformations that occur during fermentation and maturation. A gel-like texture is created following the bacterial acidification process, while color develops from the interactions between the myoglobin of the meat and nitrogen monoxide, originating from the nitrate and/or nitrite in the curing salt [9]. Lastly, the complex flavor of dry-fermented sausages is due to oxidative reactions and interactions between the sausage, meat enzymes, and microorganisms [17]. As a result, flavor development depends not only on the raw materials and processing conditions but also on the composition and metabolism of microbiota [9]. Therefore, since many variables are involved in defining the sensory profile of salami, a combined instrumental and sensory approach can be useful for highlighting the differences between samples and ensuring the authenticity of this type of traditional food. In fact, a combined instrumental and sensory approach could contribute to a more thorough evaluation of the characteristics of a food product, as well as its typicality [18].

Therefore, using a combined approach, this study aims to characterize Mora Romagnola salami and compare it with other products available on the Italian market obtained using conventional pig breeds. To the best of the authors’ knowledge, this combined instrumental and sensory approach has never been applied to salami, and this study can be considered the first application. This method could be extended to other salami, thus being universally applicable, in order to characterize and compare different types.

## 2. Materials and Methods

### 2.1. Samples

Thirteen samples were selected (Table 1): five produced using Mora Romagnola and eight manufactured with meat from modern pig breeds. Conventional samples “Contadino” (CON), “Felino” (FEL), and “Milano” (MIL) were selected as market leaders in Italy [19,20,21]. The Felino type is a protected geographical indication (PGI) from Italy, characterized by a cylindrical form with one end fatter than the other [19]. The Milano type is a cylindrical salami, initially produced in the area near Milano, and is one of the most widespread types of salami in Italy [20]. Samples CO1, CO2, CO3, CO4, and CO5 were produced with meat from conventional pig breeds and selected based on their similarity (e.g., size, weight, and diameter) to those from the Mora Romagnola breed (MO1, MO2, MO3, MO4, and MO5). The size, weight, and diameter were used to select homogeneous samples of the Mora Romagnola salami and the related conventional ones as these parameters can significantly affect the main quality traits and textural characteristics of the salami itself. Moreover, the Mora Romagnola salamis were produced by local companies by following the same process used for the corresponding conventional ones, with the only exception that 100% of the meat was from the Mora Romagnola pig breed. All samples, obtained from a single batch and directly purchased from the producers, were vacuum (99%) packed by using PE/PA 20/80 channeled vacuum bags with a water-vapor transmission of <3.5 g/m^2^/24 h and an oxygen permeability of <60 cm^3^/(m^2^ × 24 h × atm) at 23 ± 2 °C—0% r.F./RH (Orved S.p.a., Musile di Piave, VE, Italy), using a Tecnovac vacuum machine (Tecnovac, Grassobbio, BG, Italy), and stored protected from light at 4 ± 1 °C.

### 2.2. Sensory Analysis

#### 2.2.1. Participants

Descriptive analysis (DA) was performed by a panel of 12 trained judges recruited on the basis of previous involvement in sensory analysis. During their former experience in sensory analysis, the judges performed screening tests such as rankings of the perception of the basic tastes and selected olfactory attributes (e.g., rancid), to determine the adequacy of their sensory skills. 

#### 2.2.2. Evaluation Procedure

The choice of descriptors was carried out by open discussion in two preliminary sessions. These sessions were held in an area for group work. During this phase, each panelist received 4 samples for each discussion session and used visual, olfactory, taste, and texture attributes to describe them. The panel decided whether the descriptors were redundant (in which case they were removed from the profile sheet) or whether there were other attributes that should be added. As the attribute “red intensity” was not discriminant (*p* > 0.05), and the judges were not able to rate different red intensities during training, this attribute was removed from the final profile sheet. A final list of 11 attributes was defined, and descriptors were divided into olfactory (directly or orthonasally perceived, or indirectly or retronasally perceived), taste, texture, and visual descriptors (Table 2). Then, the panel members were trained on the 11 descriptors included in the profile sheet. To this end, 13 training sessions were held. The panel worked in a sensory laboratory, and each assessor worked in a single sensory booth equipped with a red light (TLD 18 w/15 red, Philips, Amsterdam, The Netherlands). The olfactory, taste, and texture evaluations were conducted under red light to prevent the judge recognizing the samples, whereas visual analysis was performed at the end of each session under white light (L 18 w/20 cool white recyclable, Osram, Munich, Germany). The panelists used reference standards during training, which were specifically formulated to help rate the intensities of the selected attributes (Table 2). Attribute intensities were rated on an unstructured 100 mm scale from 0 (not perceivable) to 100 (perceivable at the maximum level), with defined anchor points for each attribute (Table 2). Samples were presented in a randomized order (Latin square design). In each session, four different samples were considered. The samples were alphanumerically coded and presented in white plastic dishes as whole slices with a fixed thickness of 3 mm. Samples were presented monadically and evaluated individually.

Each slice of salami was served in a separate dish to avoid issues or difficulties during tasting due to the presence of more than one salami in the same dish (e.g., different flavors).

Fresh water and breadsticks were provided to the panelists between samples, to clean their mouths. The FIZZ software (Biosystèmes, Dijon, France) was used to create the profile sheet and for data collection. The final score was the average of those assigned to samples by each judge, assessed over three different sessions (i.e., in triplicate).

A performance check was carried out to confirm that the panel worked in a consistent and reliable way. The panel leader entered the assessment data and checked whether the coefficient of variation evaluated for each attribute was ≤20% by considering the mean value of the intensities given by the 12 assessors for each attribute.

### 2.3. Image Analysis

Image analysis was carried out using an “electronic eye” (Visual Analyzer VA400 IRIS, Alpha MOS, Toulouse, France), or a high-resolution (2592 × 1944 p) charge-coupled camera equipped with a specific data processing system (Alphasoft, version 14.0, Alpha MOS, Toulouse, France) and a photo camera (16 million colors). The instrument is furnished with two lights (2 × 2 fluorescent tubes) with a color temperature of 6700 °K. Samples were placed on a white plastic tray, diffusing a uniform light inside the device’s closable light chamber, and the CCD camera took a picture. The instrument was calibrated with a certified color checker (ColorChecker classic, x-Rite, Grand Rapids, MI, USA). Both image analyses (RGB scale or CIE L*a*b*) and statistical analyses were carried out. The data processing software (Alphasoft, version 14.0, Alpha MOS, Toulouse, France ) extracts color parameters from the picture.

Samples were analyzed in triplicate.

### 2.4. Volatile Compounds Analysis

Firstly, 3 g of each sample was homogenized with a mixer (IKA Werke, Staufen im Breisgau, Germany) under standardized conditions, i.e., for 30 s, of which 20 were with alternating shredding and the last 10 were continuous, to obtain a homogeneous mass. During operation, the laboratory mill was connected by silicone pipes to a water tap, to avoid overheating of the mass during shredding. Immediately after homogenization, 1 g of each sample was weighed in a 20 mL amber glass vial. Each vial was closed and conditioned at 40 °C for 2 min in the GC autosampler oven. Subsequently, a divinylbenzene/carboxen/polydimethylsiloxane (DVB/CAR/PDMS) fiber (50/30 µm, 2 cm long, from Supelco Ltd., Bellefonte, PA, USA) was exposed to the sample headspace for 30 min and immediately desorbed for 5 min at 240 °C in the GC injector, with a split ratio of 1:10. Analytes were separated on a ZB-WAX column with dimensions of 30 m × 0.25 mm ID and 1.00 µm film thickness (Phenomenex, Torrance, CA, USA). Column temperature was held at 40 °C for 10 min and then increased to 200 °C at a rate of 3 °C min^−1^. After 3 min, the temperature was increased to 240 °C at a rate of 10 °C min^−1^ and then held stable for 5 min. Helium was used as a carrier gas at a flow of 1 mL min^−1^. Volatile compounds were analyzed by quadrupolar mass-selective spectrometry (in the 30–250 amu mass range), using a GCMS-QP2010 gas chromatograph (Shimadzu Co., Kyoto, Japan) coupled with an autosampler AOC-5000 Plus (Shimadzu Co., Kyoto, Japan). Peak identification was based on a comparison of the mass spectrum data with reference spectra in the National Institute of Standards and Technology (NIST) library (2008 version), and only identifications with a match greater than 93% were taken into account. Since it was not possible to use an internal standard (due to the difficulty of homogenously solubilizing a standard molecule in a solid sample), a percentage normalization was applied: the presence of each compound was expressed as percentage with respect to the entire volatile fraction. Three replicates were performed for each sample.

### 2.5. Textural Properties

Warner–Bratzler shear testing was performed by shearing 4 × 1 × 1 cm samples with a TA-HDi Heavy Duty texture analyzer (Stable Micro Systems Ltd., Godalming, Surrey, UK) [22]. The instrument, equipped with a 25 kg loading cell and a Warner–Bratzler triangular shear blade, was set to shear the sample at a cross-head speed of 2 mm/sec. The shear force was defined as the maximum force (expressed in kg) required to shear the sample. 

A Texture Profile Analysis (TPA) was performed on a cylindrically shaped meat sample (3 cm diameter × 0.8 cm height), double compressed to 60% of its initial height. The test was performed using a TA-HDi Heavy Duty texture analyzer (Stable Micro Systems Ltd., Godalming, Surrey, UK) equipped with a 25 kg loading cell, and a cylindrical aluminum probe (5 cm diameter) was set to compress the sample at a cross-head speed of 2 mm/s (pre-test and post-test speed: 2 mm/s). The TPA parameters of hardness, cohesiveness, springiness, gumminess, and chewiness were obtained by elaborating the double compression diagram (force/deformation) [23].

A Tensile test was performed on an irregular hexagonal sample (H’ = 25 mm long × H’’ = 2 mm; 2 mm slice thickness; 5 replications/sample) according to the procedure described by Herrero et al. (2008) [24]. The tensile properties were evaluated using a TA-HDi Heavy Duty texture analyzer (Stable Micro Systems Ltd., Godalming, Surrey, UK) equipped with a 5 kg loading cell and two tensile grips. After applying a grip separation of 25 mm at a cross-head speed of 1 mm/s, the rupture force (maximum peak force required for breaking the sample, N) and the breaking strength (ratio between the rupture force and the cross-sectional area of the sample, N/cm^2^) were calculated. 

All instrumental measurements were performed at least in triplicate.

### 2.6. Statistical Analysis

The software XLSTAT version 7.5.2 (Addinsoft, Belmont, MA, USA) was used to elaborate the data using analysis of variance (ANOVA, with Fisher’s least squares difference post hoc test) and to analyze data from the chemical (volatile compounds), sensory, and textural analyses through multifactorial analysis (MFA). Pearson’s correlations were performed between sensory and instrumental data to check possible relations. Additionally, Student’s paired *t*-test was used to check for differences between the sensory results for the Mora Romagnola salami and the conventional counterparts, while one-way ANOVA was applied to the sensory values of samples CON, FEL, and MIL. The software Alphasoft version 14.0 (Alpha MOS, Toulouse, France) was used to explore data from the image analysis, using principal component analysis (PCA). In addition, the software PanelCheck (V1.4.2., GNU General Public License version 2.0 (GPLv2)) was used to monitor the panel’s performance and the training efficacy. In particular, a mixed-model three-way ANOVA was applied to evaluate the discriminant ability and repeatability of the panel, considering 3 factors (assessors, replicates, and products) and their interactions. 

## 3. Results and Discussion

### 3.1. Sensory Analysis

The reliability of the panel’s performance and the training efficiency were checked to ensure reproducibility and repeatability. The panel showed a good discrimination ability (*p* < 0.05) except for the attribute “greasiness” (*p* = 0.235). This means that for the attributes for which a statistically significant difference was found (*p* < 0.05), the panel was able to discriminate among the tested products with respect to these attributes. No replicate effect was observed (*p* < 0.001), indicating that the panel’s results were repeatable. Two significant “replicate*sample” interactions (humidity, *p* = 0.012 and fat distribution, *p* = 0.00) were probably due to chemico-physical modifications in the samples during storage between sessions. In addition, one significant (*p* < 0.05) “replicate*assessor” interactive effect (humidity, *p* = 0.49) was found, which could be related to the reference material prepared for these attributes. It would probably be necessary to also provide a reference for humidity, with lower intensity, to specifically train assessors on this attribute.

Additionally, the panel analysis and product characterization extensions of XLSTAT were used to monitor the judges’ performance during training and the effective discrimination power of each attribute.

The sensory results are discussed by comparing each Mora Romagnola product with its analogous conventional counterpart (i.e., MO1 vs. CO1, MO2 vs. CO2, and so on). 

The sensory profiling results (Table 3) showed that the intensities of the attributes *seasoning* and *fermented* for samples CO1 vs. MO1 and CO2 vs. MO2 were significantly different. For these samples, it could be useful to describe salami produced with different pig breeds, although they are not associated with a specific breed. Additionally, a significant difference related to the perception of *humidity* was also registered for MO1 compared to CO1, where the latter showed a higher intensity for this descriptor. Overall, the sensory evaluation was more useful in revealing differences among the different types of salami than in highlighting differences related to the breed. Considering the market leaders CON, FEL, and MIL, the latter had remarkably different characteristics and showed the highest intensities of *pepper*, *garlic*, *fermented, spicy*, *salty*, *humidity*, and *fat distribution* and the lowest intensities of *seasoning*, *rancid*, and *meat grain*, which is probably related to its processing [20].

**Figure 1:**
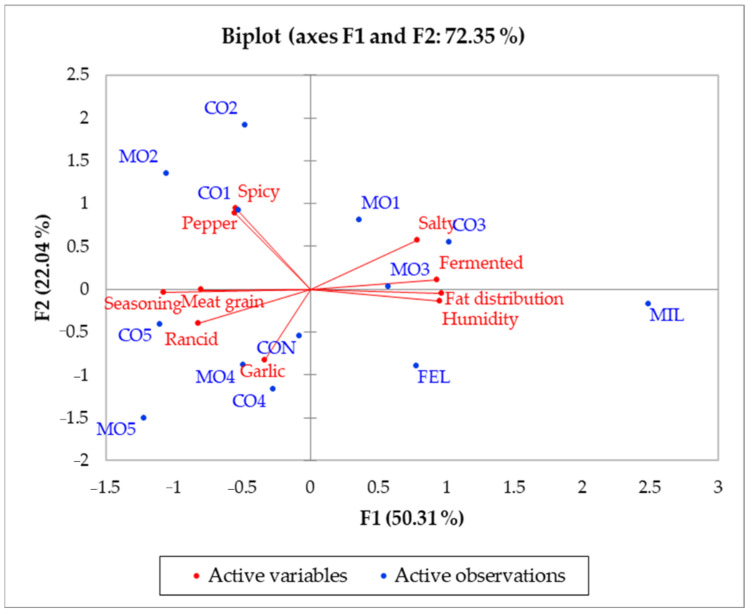
Principal component analysis (PCA) biplot for the sensory results. Samples were coded as CO1 (conventional salami with small size), MO1 (Mora Romagnola salami with the same size as CO1), CO2 (conventional salami of “Nostrano” type), MO2 (Mora Romagnola salami similar to CO2), CO3 (conventional salami of “Cacciatore” type), MO3 (Mora Romagnola salami similar to CO3), CO4 (conventional salami obtained directly from a local producer), MO4 (Mora Romagnola salami similar to CO4), CO5 (conventional salami obtained directly from a local producer), MO5 (Mora Romagnola salami similar to CO5), CON (conventional salami of “Contadino” type), FEL (conventional salami of “Felino” (PGI) type), and MIL (conventional salami of “Milano” type).

As reported in Figure 1, MIL and FEL salami had different sensory characteristics from the other samples tasted. In particular, they were characterized by the attributes of fat distribution and humidity. In addition, Mora Romagnola samples were generally in the same quadrant as their conventional counterparts, except for samples CO1 and MO1. In fact, CO1 was more characterized by pepper and spicy notes, and sample MO1 by salty taste and a fermented aroma. Our results also suggest that salty and fermented were positively correlated (r = 0.603, *p* < 0.05), while salty was negatively correlated with rancid (r = −0.640, *p* < 0.05) and seasoning (r = −0.569, *p* < 0.05) (Figure 1). In addition, humidity was negatively correlated with seasoning, which could be related to the reduction of moisture content which occurs during the ripening process in dry-cured fermented sausages [25]. Although an increase in the concentration of salt due to water loss during ripening has been reported in the literature [25], it can be assumed that the salty attribute was less perceived because it was masked by the seasoning flavors. This could explain the negative correlation (r = −0.569, *p* < 0.05) between these two attributes.

Seasoning was also positively correlated with rancid (r = 0.741, *p* < 0.05). This is probably due to the presence of several volatile compounds such as aliphatic aldehyde, which can form during the ripening of dry-cured salami and seem to contribute to flavor deterioration [26]. Additionally, a negative correlation was found between seasoning and fermented (r = −0.894, *p* < 0.05), which could be related to the decrease in several volatiles derived from microbial activities, as previously reported by Lorenzo et al. (2013) [26]. Finally, a positive correlation was reported between pepper and spicy (r = 0.872, *p* < 0.05), while a negative correlation was highlighted between meat grain and fat distribution (r = −0.854, *p* < 0.05). 

### 3.2. Image Analysis

**Figure 2:**
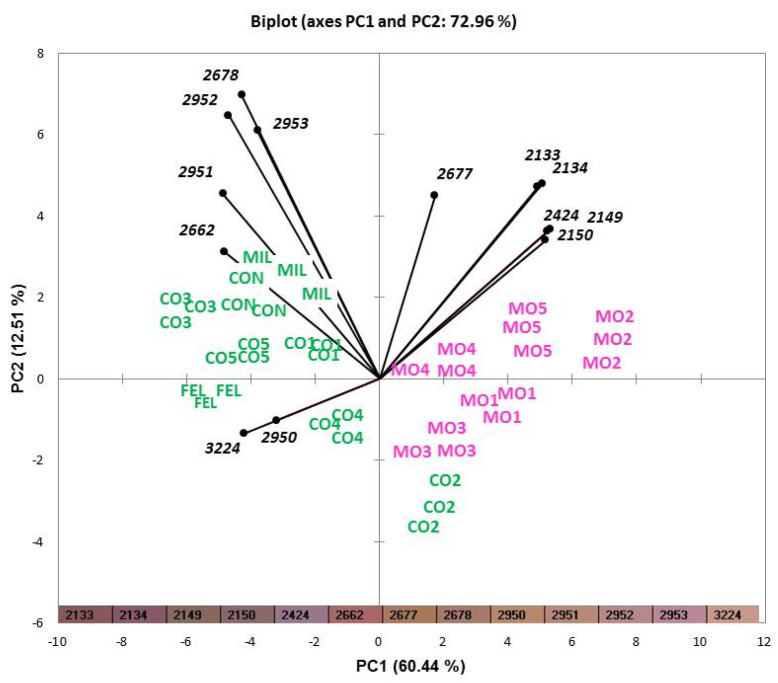
Projection of samples and variables (intensities and shades of red) on a plane (PCA). Conventional breeds and Mora Romagnola are labeled in green and pink, respectively. Samples were coded as CO1 (conventional salami with small size), MO1 (Mora Romagnola salami with the same size as CO1), CO2 (conventional salami of “Nostrano” type), MO2 (Mora Romagnola salami similar to CO2), CO3 (conventional salami of “Cacciatore” type), MO3 (Mora Romagnola salami similar to CO3), CO4 (conventional salami obtained directly from a local producer), MO4 (Mora Romagnola salami similar to CO4), CO5 (conventional salami obtained directly from a local producer), MO5 (Mora Romagnola salami similar to CO5), CON (conventional salami of “Contadino” type), FEL (conventional salami of “Felino” (PGI) type), and MIL (conventional salami of “Milano” type).

The characteristic color of salami depends on the interaction between meat pigments and products resulting from the reduction of added nitrates and nitrites. After nitrate reduction to nitrites, the latter are reduced to nitric oxide, which reacts with myoglobin, reducing it into chromogen [27]. This process improves color stability; nonetheless, salami is a very complex product in which pigment oxidation is difficult to investigate due to the numerous factors affecting the redox potential [28]. Image analysis allowed the color spectrum of each sample to be obtained in RGB coordinates (red, green, blue). The analysis is carried out rapidly, with easy-to-use equipment, thus being potentially useful also for quality control, given that no sample preparation is required. The use of a spectrophotometer tool that gives the CIE L*a*b* coordinates could be a good alternative. The color spectra were composed of more than 100 ranges of red, each identified by a specific numerical code. To evaluate its ability in discriminating the different categories of salami, data collected by electronic eye were processed by PCA. A selection of the most discriminant variables was carried out, to improve the separation between samples. Only 13 of the colors were selected according to their contribution to the two principal components (PC1 and PC2) that explained the variance (data not shown) and used as variables to perform a PCA. The projection of the samples in the factorial plane is reported in Figure 2. The different directions and locations of vectors (PCA loadings) show which variables (colors) were involved in appearance variation among the samples. The 13 colors selected allowed the two types of salami to be distinguished, highlighting the fact that the Mora Romagnola samples were darker shades of red. In fact, they are positioned in quadrant 1 (samples MO2, MO4, and MO5 are in the upper-right quadrant) or in quadrant 2 (samples MO1 and MO3 are positioned in the lower-right quadrant), while conventional salami are positioned in quadrants 3 and 4, with the exception of sample CO2, which was characterized by a similar color to samples MO3 and MO1.

In particular, different red intensities were mainly associated with the pig breed used for their production. The application of the software available with the instrument (Alphasoft, version 14.0, Alpha MOS, Toulouse, France) allowed color spectra to be grouped in a range of 16 bits for each RGB coordinate, resulting in 4096 variables, reported by the software as histograms. The proportion of each color in the analyzed image, on a fixed scale of 4096 colors, is represented as a percentage. Specifically, variables such as “color 2424” (L* = 53.967, a* = 14.881, b* = −3.739), “color 2133” (L* = 42.862, a* = 20.415, b* = 8.624), “color 2134” (L* = 43.231, a* = 22.142, b* = −0.588), “color 2150” (L* = 47.289, a* = 13.213, b* = 5.207), “color 2677” (L* = 54.833, a* = 16.479, b* = 25.386), and “color 2149” (L* = 46.966, a* = 11.485, b* = 14.237), associated with the strongest red intensities, characterized several Mora Romagnola samples. On the other hand, “color 2951” (L* = 61.180, a* = 17.318, b* = 16.183), “color 2678” (L* = 55.089, a* = 17.797, b* = 16.653), “color 3224” (L* = 61.167, a* = 16.898, b* = 15.778), “color 2953” (L* = 61.842, a* = 20.720, b* = −0.973), “color 2952” (L* = 61.487, a* = 18.914, b* = 7.584), “color 2662” (L* = 51.239, a* = 26.376, b* = 11.415) and “color 2950” (L* = 60.917, a* = 15.939, b* = 24.752) were found in conventional salami.

This is an important result: although consumer expectation is that there should be different red intensities according to pig breed, the trained judges declared that they were not able to observe differences in red intensities among the samples. Thus, the use of an electronic eye could support visual evaluation of red intensity in salami and could be useful for discriminating salami produced with Mora Romagnola from that produced with other breeds.

### 3.3. Volatile Compounds Analysis

It is well known that many enzymatic and non-enzymatic reactions occur during the fermentation and aging of dry-cured meat products, such as proteolysis and lipolysis, along with protein and lipid oxidation, Maillard reactions, and Strecker degradation. These changes give rise to the formation of volatile compounds [29]. In this investigation, 33 volatile compounds were tentatively identified (Table 4). Low percentages of ketones were detected, with the exception of 2-butanone in sample MO3 (12.76%); conversely, this volatile was not detected in the CO3 conventional counterpart. This molecule is already known to be one of the contributors to an “apricot” note in sausages [30]. The most represented alcohols were ethanol and 2-butanol. In particular, ethanol was detected in all samples produced with Mora Romagnola, with higher percentages than in conventional samples. This compound can derive from the reduction of aldehydes formed by lipid metabolism [31] or from microbial fermentation [32]. Pentanol, hexanol, and 1-octen-3-ol are also produced in the same way; the latter is considered to be responsible for a mushroom-like odor [33] and is produced during degradation of lipid hydroperoxides [34]. The aging process of dry-cured salami is known to have an important role in the formation of aldehydes [25]. Among these, hexanal was the most represented. Hexanal is known to impart green, floral, or even grassy notes [35,36]. High percentages of hexanal were recorded for samples FEL (72.58%), CON (46.86%), MIL (31.56%), and CO5 (44.78%); conversely, it was not detected in MO5. The second most abundant aldehyde was phenylacetaldehyde, with a high percentage (38.07%) in MO4. All other samples showed low levels of this molecule, which is an aroma-active compound formed from phenylalanine through the Strecker degradation pathway [37]. Allyl methyl sulfide is responsible for garlic notes and is naturally present in many plants, e.g., alliums such as garlic, onion, and leek, which are normally added during salami production [38]. This compound was detected in samples MO3, MO4, and MO5, as well as in their conventional counterparts (low amounts in MO1 and MO2). In accordance with previous studies [25], the chemical class of terpenes was the most represented, and the presence of these compounds is probably due to the addition of spices. For example, α-pinene, identified in all samples except FEL, is a terpene found in coniferous and rosemary essential oils [39]. The isomers of α-phellandrene and β-phellandrene are specific to certain plants: the first is typical of *Eucalyptus phellandra*, while the latter has been frequently found in essential fennel oil [40]. In addition, 3-carene, a molecule linked to pepper scents, was mainly found in samples MO4, MO5, and their conventional counterparts produced with the same recipe, while limonene was the most represented terpene and can be associated with pine/peppery/lemon sensory notes [41]. It is interesting to highlight that sample FEL did not contain terpenes. This could be related to its production process and recipe. Finally, several acids were identified. Butanoic acid is responsible for the perception of rancidity in food [36]; it was detected only in samples MO1, CO3, and in the market leader sample MIL. The most abundant acid was acetic acid, which showed the highest percentage in the MIL sample (10.79%). Although several volatile compounds were different in some of the samples, a trend in their concentrations that was clearly related to the different type of meat used (Mora Romagnola vs. conventional) was not observed. On the other hand, the samples available on the market showed remarkable differences in terms of their aromatic profile. This result could be associated with the different production techniques and use of spices. In this context, the market leader products were characterized by a less complex volatile compounds profile: sample FEL exhibited an absence of terpenes, and ethanol was not detected in CON, FEL, or MIL. Furthermore, Kim et al. (2021) [42] highlighted a low presence of terpenes in Felino salami in comparison with others such as Saucisse and Napoli salami. 

On the other hand, substantial variability was observed among the Mora Romagnola samples, which may be related to the different curing times and processing methods. This is not an unexpected result, since salami produced with this local breed are traditional products, whose recipes (specific ingredients) and handcrafted preparation are characterized by a certain variability and are linkable to the concept of local food [2,3].

### 3.4. Combining Volatile Compounds and Sensory Data

**Figure 3:**
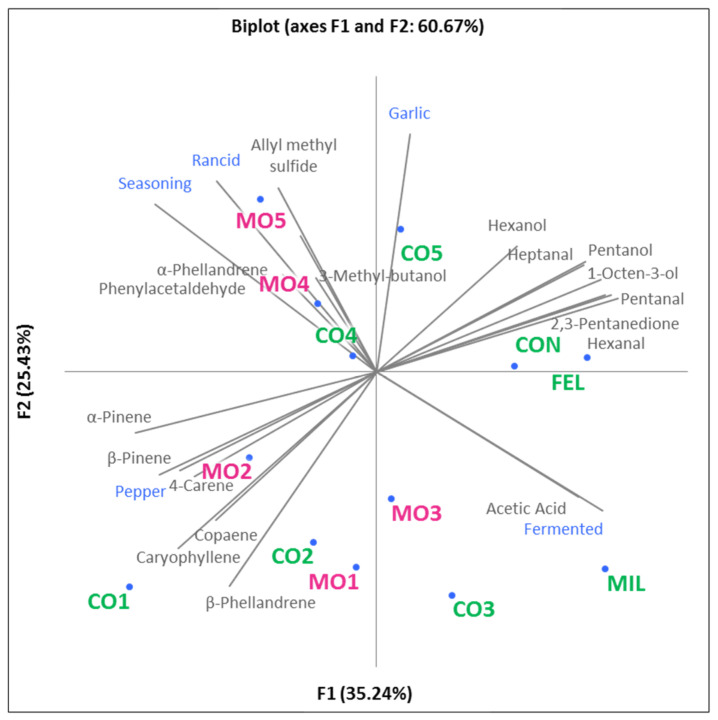
Projection of samples and variables (intensity and shades of red) on a plane (PCA). Samples from conventional breeds and Mora Romagnola samples are labeled in green and pink, respectively. Samples were coded as CO1 (conventional salami with small size), MO1 (Mora Romagnola salami with the same size as CO1), CO2 (conventional salami of “Nostrano” type), MO2 (Mora Romagnola salami similar to CO2), CO3 (conventional salami of “Cacciatore” type), MO3 (Mora Romagnola salami similar to CO3), CO4 (conventional salami obtained directly from a local producer), MO4 (Mora Romagnola salami similar to CO4), CO5 (conventional salami obtained directly from a local producer), MO5 (Mora Romagnola salami similar to CO5), CON (conventional salami of “Contadino” type), FEL (conventional salami of “Felino” (PGI) type), and MIL (conventional salami of “Milano” type).

Sensory results and contents of volatile compounds were analyzed by multifactorial analysis also to look for correlations. In particular, 18 of the 33 molecules and 5 of the 11 sensory attributes were selected according to their discrimination power and the sensory notes that they are known to be responsible for. The MFA biplot is shown in Figure 3. The first two components were responsible for 60.67% of the total variance.

Samples MO4, MO5, CO4, and CO5 were similar and were described by the attributes seasoning and rancid, and by volatile compounds related to oxidation (phenylacetaldehyde) and garlic (allyl methyl sulfide). The attributes seasoning and rancid were positively correlated (r = 0.75); thus, it can be hypothesized that an increased intensity of seasoning could be linked to a stronger perception of rancidity.

Samples MO1, MO2, CO1, and CO2 were characterized by the presence of many terpenes and by the sensory attribute of pepper. This latter descriptor was positively correlated with volatile compounds such as caryophyllene, β-phellandrene, and α-pinene. This finding is in agreement with previous studies on MIL salami [35]. Samples MO3 and CO3 were characterized by the presence of acetic acid and the sensory perception of the attribute fermented, which were positively correlated (r = 0.69). This result is in accordance with the investigation by Stankhe et al. on dried sausages [36].

### 3.5. Textural Properties

The findings for textural properties assessed using Warner–Bratzler testing, TPA, and tensile testing are reported in Table 5. Considering the entire set of samples, MIL displayed remarkably different textural properties. Indeed, it exhibited the lowest values for all textural parameters. In contrast, FEL and CON revealed textural profiles comparable to those of the other 10 samples. In this context, since the conventional samples were selected according to their similarity to their Mora Romagnola counterparts, the results for textural properties will be discussed by considering the samples as pairs. CO1 and MO1 exhibited analogous values for almost all parameters. Similarly, only negligible dissimilarities (not displaying any clear trends) were observed when comparing the textural properties of CO4 vs. MO4. The main differences were found between CO2 vs. MO2, CO3 vs. MO3, and CO5 vs. MO5.

In detail, compared to their conventional counterparts (CO2 and CO3), significantly higher hardness and hardness-related parameters (chewiness and gumminess) were found in MO2 and MO3, which also exhibited increased cohesiveness. In addition, remarkably higher springiness and rupture force (+48%) were observed in MO2 compared to its conventional counterpart. Similarly, remarkably higher springiness (+16%) and chewiness (+40%) were observed in MO5 compared to its conventional counterpart. In addition, MO5 exhibited the highest breaking strength. These findings are in agreement with those obtained in a previous study performed by Saccani et al. (2013) [43], and clearly show that products exhibiting different textural properties are present on the market. Among the variables, both fermentation and aging period can influence the textural properties of dry-fermented sausages to a remarkable degree [43]. The higher values exhibited by samples CO5 and MO5 for all the parameters considered might be the result of a longer curing time which, as a direct consequence of a higher water loss through evaporation, may have resulted in the development of firmer sausages [44]. Indeed, hardness, chewiness, and gumminess were found to increase over the aging period as a consequence of protein coagulation at low pH values which, in turn, results in protein denaturation and reduces the water-holding ability of the meat, leading to a decrease in moisture content [43]. Additionally, even the starter culture added in the formulation may have played a role in defining the physicochemical and textural traits of the final product [45,46,47]. Indeed, by affecting the rate and extent of pH decline following lactic fermentation, starter cultures may affect proteolysis to a remarkable degree [45,48], as well as solubilization and the subsequent gelification of the myofibrillar proteins responsible for gel formation [49].

### 3.6. Combining Data from Texture Analysis and Sensory Attributes

**Figure 4:**
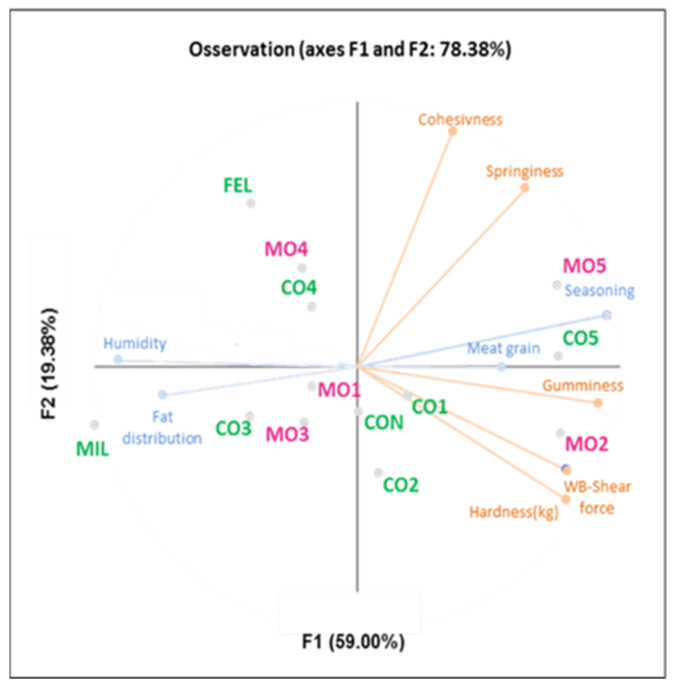
Projection of samples and variables (textural and sensory results) in multifactorial analysis (MFA biplot). Mora Romagnola and conventional pig breed salami samples are labelled in pink and green, respectively. Samples were coded as CO1 (conventional salami with small size), MO1 (Mora Romagnola salami with the same size as CO1), CO2 (conventional salami of “Nostrano” type), MO2 (Mora Romagnola salami similar to CO2), CO3 (conventional salami of “Cacciatore” type), MO3 (Mora Romagnola salami similar to CO3), CO4 (conventional salami obtained directly from a local producer), MO4 (Mora Romagnola salami similar to CO4), CO5 (conventional salami obtained directly from a local producer), MO5 (Mora Romagnola salami similar to CO5), CON (conventional salami of “Contadino” type), FEL (conventional salami of “Felino” (PGI) type), and MIL (conventional salami of “Milano” type).

The results for textural properties and sensory evaluation were analyzed using multifactorial analysis (Figure 4). In particular, four sensory attributes and six textural parameters were selected according to their contribution to the two principal components (PC1 and PC2) that explained the variance. The first two components explained 78.38% of the total variance. The findings showed that MO5 was similar to its conventional counterpart (CO5), and both were close in the attributes of seasoning and meat grain and the textural parameter of gumminess. This textural property, in turn, was positively correlated with the attribute of seasoning (r = 0.80). This trend could be partly explained by considering the aforementioned increase in hardness and chewiness during curing [43,50]. Thus, a longer curing period might account for the increased perception of the seasoning attribute by the panel. Accordingly, the Mora Romagnola sample MO2 was close to the vectors related to the textural parameters of WB shear force, hardness, and gumminess (Figure 4).

Samples MO4 and CO4 were characterized by the attribute of humidity; consequently, as shown in Table 5, MO4 and CO4 exhibited low hardness and WB shear force values (MO4: 79.46 and 10.10 N; CO4: 140.13 and 17.85 N). Hence, the higher the humidity, the lower the toughness (instrumentally assessed). The pairs CO1/MO1 and CO2/MO2 also appeared to be similar and were characterized by similar hardness and WB shear force.

Samples CO3 and MO3 were characterized by high intensities of fat distribution and consequently by low values of cohesiveness and springiness. Indeed, a higher homogeneity of fat distribution perceived by the panel corresponded to a lower energy being needed to irreversibly deform the sample structure. Considering these results, differences could be related to the production system and the different salami types. These differences did not seem to be ascribable to the pig breed: thus, standardization of specific technological attributes related to manufacturing of Mora Romagnola salami could be useful for making this product distinguishable from others present in the Italian market. To confirm these results, this first study should be replicated by sampling a higher number of batches of each salami.

## 4. Conclusions

The identification of markers for the discrimination of salami types currently available on the market and produced with different pig breeds is nowadays very relevant. One problem is that it is difficult to identify markers which are able to support this discrimination. Herein, olfactory and taste attributes were used as sensory levers, showing great variability between samples. This variability was not directly ascribable to the breed of pig, and the sensory characteristics were linked to the production system, as samples obtained with similar technological approaches had similar aromatic and textural profiles. Specifically, 33 volatile molecules mainly belonging to the chemical classes of terpenes, aldehydes, alcohols, ketones, and acids were detected, but no markers were identified that could differentiate Mora Romagnola salami from conventional products. Moreover, a substantial variability was found in their concentrations among the Mora Romagnola samples. These differences appeared to be linked to the various processing methods. Standardization of specific technological parameters related to the manufacturing of Mora Romagnola salami could be promoted, to help customers to identify this typical Italian salami, thus contributing to making it distinguishable from others present in the Italian and global markets. Color differences among Mora Romagnola and conventional salami were unperceivable by humans; nevertheless, an electronic eye allowed to discriminate between samples produced with different pig breeds. Salami produced with Mora Romagnola and conventional breeds were not discriminated by instrumental texture analysis. Indeed, other factors such as the recipe, dimension, and curing time can be responsible for the large variability in textural properties among all samples. 

This investigation showed that the traditional craftsmanship used in its typical production leads to variability in the characteristics of Mora Romagnola salami, which is related to the concept of local food. Furthermore, samples appeared to be distinguished according to the technology used for production rather than by the pig breed. To confirm this, an investigation with a dedicated sampling and experimental plan should be carried out. This study highlighted the large variability among the salami analyzed. Such differences did not seem to be specifically related to the Mora Romagnola breed, with the single exception of the red color intensities evaluated by the electronic eye. In this latter case, samples produced with this extensively farmed pig breed appeared to be characterized by stronger red intensities. Indeed, this investigation allowed the identification of several quality traits. Further analytical efforts should be made towards a definition of markers (e.g., genetic traits) that could potentially be included in a protocol that describes and protects the unique characteristics of this product. This is highly desirable to ensure its authenticity. A further investigation considering a higher number of batches of each salami will be useful to confirm the preliminary outcomes of this study. In addition, applying the same multidisciplinary approach to different types of salami/fermented sausages might be of interest, to provide an extensive characterization of the products available in the Italian market.

Producers are therefore advised to develop a standardized production protocol for Mora Romagnola dry-cured salami, in order to provide strong recognition for these products on the market. Indeed, the production of typical dry-cured and dry-fermented products using local breeds can contribute to preserve the genetic diversity of the animal species itself, which, being strongly related to the distinctive nutritional profile of the meat, may play a relevant role in ensuring the achievement of a biodiversity to cover the nutritional requirements of the population.

## Figures and Tables

**Table 1:** Characteristics of salami samples.

Sample	Characteristics
CO1	Conventional salami with small size (50 g)
MO1	Mora Romagnola salami with the same size as CO1 (50 g)
	
CO2	Conventional salami of “Nostrano” type (500 g)
MO2	Mora Romagnola salami similar to CO2 (500 g)
	
CO3	Conventional salami of “Cacciatore” type (260 g)
MO3	Mora Romagnola salami similar to CO3 (260 g)
	
CO4	Conventional salami (300 g)
MO4	Mora Romagnola salami similar to CO4 (300 g)
	
CO5	Conventional salami (300 g)
MO5	Mora Romagnola salami similar to CO5 (300 g)
	
CON	Conventional salami of “Contadino” type (550 g) [21]
FEL	Conventional salami of “Felino” (PGI) type (1000 g) [19,21]
MIL	Conventional salami of “Milano” type (1000 g) [20,21]

**Table 2:** Attributes used in the profile sheet for salami samples and their definitions, with anchor points and references used during the training phase.

Attribute	Definition	Reference Materials	Anchor Points for Reference Materials (Intensity)
*Direct/indirect olfactory attributes*			
**Seasoning**	Direct/indirect olfactory note, reminiscent of seasoned meat	Minced pork meat (fresh) and sample CO1 (seasoned)	Fresh: 20; Seasoned: 80
**Pepper**	Direct/indirect olfactory note, reminiscent of pepper	4 g of pepper in 20 g of minced pork meat	100
**Garlic**	Direct/indirect olfactory note, reminiscent of garlic	2 g of garlic powder in 20 g of minced pork meat	100
**Rancid**	Direct/indirect olfactory note linkable to oxidized/old food	CO1 samples that had undergone forced oxidation for 7 days	100
**Fermented**	Pungent olfactory note, reminiscent of acetic acid	“Golfetta” type of pork salami	100
*Taste attributes*			
**Spicy**	Mouth inflammatory effect	/	
**Salty**	Salty elementary taste	/	
*Texture attributes*			
**Greasiness**	Fatty/greasy sensation perceived while biting	“Milano” type of pork salami	80/100
**Humidity**	Juiciness released by sample while biting	“Golfetta” type of pork salami	80/100
*Visual attributes*			
**Meat grain**	Lean part evaluation: lean meat distribution in the slice	/	
**Fat distribution**	Fat part evaluation: fat part distribution in the slice	/	

**Table 3:** Sensory results for salami samples. Mean values ± standard deviations of sensory data.

Sample	Seasoning	Pepper	Garlic	Rancid	Fermented	Spicy	Salty	Humidity	Meat Grain	Fat Distribution
CO1	61 ± 6	37 ± 7	16 ± 3	4 ± 2	0 ± 0	18 ± 3	31 ± 3	23 ± 3	28 ± 7	57 ± 18
MO1	44 * ± 16	28 ± 7	19 ± 5	2 ± 1	5 * ± 1	20 ± 1	33 ± 1	36 * ± 10	32 ± 4	45 ± 9
CO2	49 ± 2	42 ± 5	19 ± 2	3 ± 2	6 ± 1	26 ± 3	33 ± 2	26 ± 3	44 ± 0	36 ± 1
MO2	65 * ± 4	36 ± 4	18 ± 7	3 ± 1	1 * ± 1	22 ± 1	33 ± 7	19 ± 8	43 ± 13	29 ± 5
CO3	38 ± 4	21 ± 4	19 ± 1	1 ± 1	8 ± 2	17 ± 6	35 ± 8	30 ± 2	31 ± 2	59 ± 7
MO3	41 ± 4	22 ± 5	18 ± 6	2 ± 2	5 ± 2	15 ± 5	31 ± 2	42 ± 8	43 ± 10	55 ± 12
CO4	44 ± 3	23 ± 6	25 ± 10	4 ± 3	4 ± 1	13 ± 4	25 ± 3	38 ± 7	40 ± 3	34 ± 8
MO4	56 ± 6	28 ± 3	29 ± 8	2 ± 2	2 ± 3	14 ± 4	27 ± 7	32 ± 7	44 ± 11	46 ± 4
CO5	67 ± 2	26 ± 3	27 ± 11	4 ± 1	1 ± 0	18 ± 10	30 ± 4	17 ± 4	39 ± 3	36 ± 7
MO5	69 ± 2	19 ± 5	27 ± 1	7 ± 8	2 ± 3	14 ± 4	27 ± 3	15 ± 1	37 ± 2	42 ± 6
^1^ CON	48 ^a^ ± 5	23 ^a^ ± 6	27 ^a^ ± 3	2 ^a^ ± 1	5 ^a^ ± 2	13 ^a^ ± 1	33 ^a^ ± 4	28 ^b^ ± 1	49 ^a^ ± 10	37 ^b^ ± 10
^1^ FEL	43 ^a^ ± 13	13 ^b^ ± 2	19 ^b^ ± 3	4 ^a^ ± 2	5 ^a^ ± 2	9 ^b^ ± 1	34 ^a^ ± 6	37 ^a^ ± 9	30 ^b^ ± 9	52 ^b^ ± 13
^1^ MIL	23 ^b^ ± 4	19 ^a^ ± 3	22 ^a^ ± 5	0 ^b^ ± 0	8 ^a^ ± 1	10 ^a^ ± 3	38 ^a^ ± 4	46 ^a^ ± 4	11 ^c^ ± 2	89 ^a^ ± 3

Values for Mora Romagnola samples (MO1–MO5) followed by * significantly differ (Student’s paired *t*-test) from their conventional-breed counterparts. In addition, values for market leader samples ^(1)^ followed by different letters significantly differed among these samples (one-way ANOVA, Tukey-HSD, *p* < 0.05). Samples were coded as CO1 (conventional salami with small size), MO1 (Mora Romagnola salami with the same size as CO1), CO2 (conventional salami of “Nostrano” type), MO2 (Mora Romagnola salami similar to CO2), CO3 (conventional salami of “Cacciatore” type), MO3 (Mora Romagnola salami similar to CO3), CO4 (conventional salami obtained directly from a local producer), MO4 (Mora Romagnola salami similar to CO4), CO5 (conventional salami obtained directly from a local producer), MO5 (Mora Romagnola salami similar to CO5), CON (conventional salami of “Contadino” type), FEL (conventional salami of “Felino” (PGI) type), and MIL (conventional salami of “Milano” type).

**Table 4:** (**a**) Volatile compounds (relative area % and standard deviations) detected in conventional (CO1, CO2, and CO3,) and Mora Romagnola (MO1, MO2, and MO3) samples. Compounds are listed by chemical classes. Samples were coded as CO1 (conventional salami with small size), MO1 (Mora Romagnola salami with the same size as CO1), CO2 (conventional salami of “Nostrano” type), MO2 (Mora Romagnola salami similar to CO2), CO3 (conventional salami of “Cacciatore” type) and MO3 (Mora Romagnola salami similar to CO3). (**b**) Volatile compounds (relative area % and standard deviations) detected in conventional (CO4 and CO5), Mora Romagnola (MO4 and MO5), and CON, FEL, and MIL salami samples. Compounds are listed by chemical classes. Samples were coded as CO4 (conventional salami obtained directly from a local producer), MO4 (Mora Romagnola salami similar to CO4), CO5 (conventional salami obtained directly from a local producer), MO5 (Mora Romagnola salami similar to CO5), CON (conventional salami of “Contadino” type), FEL (conventional salami of “Felino” (PGI) type), and MIL (conventional salami of “Milano” type).

(a)												
Pubchem CID		Sample	CO1			MO1			CO2	MO2	CO3	MO3
	Compound											
***Ketones***												
5324275	2-Butanone		n.d.			0.49 ± 0.11			n.d.	3.79 ± 0.50	n.d.	12.76 ± 1.57
11747	2,3-Pentanedione		n.d.			n.d.			n.d.	n.d.	n.d.	n.d.
179	3-Hydroxy-2-butanone		0.95 ± 0.01			0.77 ± 0.02			0.75 ± 0.04	2.20 ± 0.19	8.85 ± 0.89	4.39 ± 0.37
***Alcohols***												
702	Ethanol		1.43 ± 0.09			24.62 ± 1.93			2.31 ± 0.74	7.16 ± 0.23	1.83 ± 0.23	19.63 ± 1.69
6568	2-Butanol		n.d.			0.29 ± 0.06			n.d.	4.95 ± 0.24	n.d.	23.56 ± 1.78
6276	1-Pentanol		n.d.			n.d.			n.d.	n.d.	n.d.	n.d.
31260	3-Methyl-1-butanol		n.d.			n.d.			n.d.	0.43 ± 0.02	n.d.	2.02 ± 0.50
8103	1-Hexanol		n.d.			n.d.			n.d.	n.d.	n.d.	n.d.
18827	1-Octen-3-ol		n.d.			n.d.			n.d.	n.d.	n.d.	n.d.
***Aldehydes***												
8063	Pentanal		n.d.			n.d.			n.d.	n.d.	n.d.	n.d.
6184	Hexanal		0.07 ± 0.01			1.23 ± 0.20			n.d.	n.d.	20.02 ± 1.77	2.14 ± 0.39
8130	Heptanal		n.d.			n.d.			n.d.	n.d.	n.d.	n.d.
31289	Nonanal		n.d.			n.d.			n.d.	n.d.	n.d.	n.d.
5283324	2-Octenal		n.d.			n.d.			n.d.	n.d.	n.d.	n.d.
240	Benzaldehyde		n.d.			n.d.			n.d.	n.d.	n.d.	n.d.
998	Phenylacetaldehyde		n.d.			2.54 ± 0.28			n.d.	6.80 ± 0.63	n.d.	8.10 ± 1.25
***Acids***												
176	Acetic Acid		n.d.			n.d.			1.17 ± 0.32	1.51 ± 0.07	7.69 ± 0.74	n.d.
1032	Propionic Acid		n.d.			n.d.			0.10 ± 0.09	0.97 ± 0.02	n.d.	n.d.
264	Butanoic Acid		n.d.			1.07 ± 0.11			n.d.	n.d.	0.62 ± 0.09	n.d.
10430	3-Methylbutanoic Acid		0.80 ± 0.26			n.d.			0.54 ± 0.11	0.65 ± 0.17	n.d.	n.d.
***Terpenes***												
6654	α-Pinene		13.56 ± 1.13			5.16 ± 0.48			8.43 ± 0.61	7.38 ± 0.37	9.27 ± 0.84	4.37 ± 0.60
7460	α-Phelladrene		n.d.			n.d.			4.48 ± 0.49	3.53 ± 0.05	2.51 ± 0.22	n.d.
14896	β-Pinene		26.58 ± 1.42			12.28 ± 0.69			12.29 ± 0.55	n.d.	n.d.	7.73 ± 1.14
11142	β-Phellandrene		27.96 ± 2.41			15.02 ± 0.24			6.52 ± 0.09	18.05 ± 0.42	14.48 ± 1.75	7.52 ± 0.77
26049	3-Carene		2.45 ± 0.04			n.d.			19.19 ± 0.41	0.21 ± 0.18	0.36 ± 0.07	3.79 ± 0.72
7462	α-Terpinene		0.34 ± 0.03			0.22 ± 0.01			1.66 ± 2.71	0.45 ± 0.04	n.d.	n.d.
440917	D-Limonene		0.11 ± 0.02			25.77 ± 1.76			36.44 ± 2.35	31.14 ± 0.19	33.53 ± 4.79	6.69 ± 0.85
530422	4-Carene		4.00 ± 0.26			n.d.			0.28 ± 0.03	0.23 ± 0.03	n.d.	n.d.
12303902	Copaene		3.18 ± 1.11			1.28 ± 0.20			n.d.	n.d.	0.70 ± 0.19	n.d.
5281515	Caryophyllene		13.44 ± 3.48			5.52 ± 0.84			3.25 ± 0.30	4.83 ± 0.43	4.90 ± 0.52	4.13 ± 0.35
***Others***												
66282	Allyl methyl sulfide		n.d.			0.17 ± 0.15			n.d.	0.88 ± 0.32	1.49 ± 0.15	1.86 ± 0.25
1140	Methylbenzene		4.57 ± 0.58			2.45 ± 0.13			0.99 ± 0.07	n.d.	n.d.	0.74 ± 0.66
76451	Vinyl caproate		n.d.			0.23 ± 0.05			n.d.	n.d.	n.d.	n.d.
**(b)**												
**Pubchem CID**		**Sample**	**CO4**		**MO4**		**CO5**		**MO5**	**CON**	**FEL**	**MIL**
	**Compound**											
***Ketones***												
5324275	2-Butanone		2.25 ± 0.46		1.49 ± 0.91		0.47 ± 0.43		1.07 ± 0.14	n.d.	n.d.	3.92 ± 0.52
11747	2,3-Pentanedione		n.d.		n.d.		1.05 ± 0.03		n.d.	0.69 ± 0.04	1.39 ± 0.10	1.02 ± 0.02
179	3-Hydroxy-2-butanone		0.94 ± 0.06		n.d.		n.d.		0.84 ± 0.21	2.48 ± 0.12	n.d.	2.37 ± 0.20
***Alcohols***												
702	Ethanol		n.d.		4.66 ± 1.74		n.d.		13.12 ± 0.78	n.d.	n.d.	n.d.
6568	2-Butanol		1.80 ± 0.14		1.62 ± 0.56		n.d.		1.28 ± 0.20	n.d.	0.67 ± 0.06	3.88 ± 0.47
6276	1-Pentanol		n.d.		n.d.		2.11 ± 0.03		n.d.	2.22 ± 0.07	2.06 ± 0.20	1.12 ± 0.05
31260	3-Methyl-1-butanol		1.42 ± 0.06		1.25 ± 0.31		0.63 ± 0.12		1.24 ± 0.53	n.d.	n.d.	n.d.
8103	1-Hexanol		n.d.		n.d.		2.29 ± 0.38		n.d.	2.34 ± 0.22	0.73 ± 0.07	0.51 ± 0.02
18827	1-Octen-3-ol		n.d.		n.d.		1.04 ± 0.11		0.27 ± 0.09	0.92 ± 0.04	2.31 ± 0.29	1.05 ± 0.12
***Aldehydes***												
8063	Pentanal		n.d.		n.d.		1.37 ± 0.07		n.d.	3.09 ± 0.22	3.90 ± 0.09	1.49 ± 0.01
6184	Hexanal		0.26 ± 0.04		n.d.		44.78 ± 1.69		0.77 ± 0.23	46.86 ± 1.27	72.58 ± 2.31	31.56 ± 0.85
8130	Heptanal		n.d.		n.d.		0.70 ± 0.04		n.d.	1.01 ± 0.06	0.90 ± 0.21	0.34 ± 0.04
31289	Nonanal		n.d.		n.d.		n.d.		n.d.	0.50 ± 0.06	n.d.	n.d.
5283324	2-Octenal		n.d.		n.d.		n.d.		n.d.	0.36 ± 0.05	n.d.	n.d.
240	Benzaldehyde		0.60 ± 0.16		1.60 ± 0.52		0.93 ± 0.02		n.d.	n.d.	0.67 ± 0.07	n.d.
998	Phenylacetaldehyde		4.07 ± 1.53		38.07 ± 9.66		n.d.		4.59 ± 2.49	n.d.	2.87 ± 0.94	n.d.
***Acids***												
176	Acetic Acid		n.d.		n.d.		n.d.		n.d.	0.21 ± 0.03	4.72 ± 1.79	10.79 ± 0.51
1032	Propionic Acid		n.d.		n.d.		n.d.		n.d.	n.d.	n.d.	n.d.
264	Butanoic Acid		n.d.		n.d.		n.d.		n.d.	n.d.	n.d.	0.29 ± 0.02
10430	3-Methylbutanoic Acid		n.d.		n.d.		0.38 ± 0.11		n.d.	n.d.	n.d.	1.14 ± 0.05
***Terpenes***												
6654	α-Pinene		9.18 ± 0.54		5.41 ± 1.24		5.94 ± 0.58		8.73 ± 0.22	5.61 ± 0.41	n.d.	3.72 ± 0.05
7460	α-Phelladrene		7.23 ± 0.36		3.80 ± 0.22		n.d.		6.05 ± 0.37	1.30 ± 0.17	n.d.	1.38 ± 0.04
14896	β-Pinene		1.64 ± 1.27		7.12 ± 1.28		1.46 ± 0.05		11.06 ± 0.29	3.16 ± 0.30	n.d.	5.54 ± 0.24
11142	β-Phellandrene		1.77 ± 0.06		1.11 ± 0.14		n.d.		1.22 ± 0.02	7.96 ± 0.62	n.d.	7.38 ± 0.33
26049	3-Carene		26.61 ± 1.26		15.97 ± 1.67		15.66 ± 1.04		24.26 ± 1.23	n.d.	n.d.	7.10 ± 0.28
7462	α-Terpinene		n.d.		n.d.		n.d.		n.d.	n.d.	n.d.	
440917	D-Limonene		19.68 ± 0.84		10.12 ± 0.23		10.48 ± 0.74		16.93 ± 0.99	15.36 ± 1.00	n.d.	8.64 ± 0.32
530422	4-Carene		n.d.		n.d.		n.d.		n.d.	n.d.	n.d.	n.d.
12303902	Copaene		n.d.		n.d.		n.d.		n.d.	n.d.	n.d.	n.d.
5281515	Caryophyllene		1.51 ± 0.26		0.88 ± 0.21		1.23 ± 0.59		1.74 ± 0.18	2.38 ± 0.19	n.d.	1.23 ± 0.16
***Others***												
66282	Allyl methyl sulfide		4.02 ± 0.88		4.33 ± 1.75		1.22 ± 0.54		5.43 ± 0.48	n.d.	n.d.	n.d.
1140	Methylbenzene		n.d.		n.d.		n.d.		n.d.	n.d.	n.d.	n.d.
76451	Vinyl caproate		n.d.		n.d.		2.11 ± 1.32		n.d.	0.93 ± 0.14	n.d.	1.15 ± 0.10

n.d.: not detected.

**Table 5:** Mean values of textural parameters assessed in salami manufactured from pigs belonging to conventional or Mora Romagnola breeds. Samples were coded as CO1 (conventional salami with small size), MO1 (Mora Romagnola salami with the same size as CO1), CO2 (conventional salami of “Nostrano” type), MO2 (Mora Romagnola salami similar to CO2), CO3 (conventional salami of “Cacciatore” type), MO3 (Mora Romagnola salami similar to CO3), CO4 (conventional salami obtained directly from a local producer), MO4 (Mora Romagnola salami similar to CO4), CO5 (conventional salami obtained directly from a local producer), MO5 (Mora Romagnola salami similar to CO5), CON (conventional salami of “Contadino” type), FEL (conventional salami of “Felino” (PGI) type), and MIL (conventional salami of “Milano” type).

	Parameter	WB Shear Force (N)	Hardness (N)	Cohesiveness	Gumminess (N)	Springiness	Chewiness (N)	Rupture Force (N)	Breaking Strength (N × cm^2^)
Sample									
**CO1**		33.55 ± 14.32	226.61 ± 28.45	2.71 ± 0.20	617.05 ± 108.89	2.27 ± 0.26	1414.60 ± 361.01	2.16 ± 0.98	0.88 ± 0.39
**MO1**		21.78 * ± 6.67	215.82 ± 69.65	2.50 ± 0.29	549.36 ± 222.70	2.22 ± 0.25	1260.59 ± 633.73	1.57 ± 1.08	0.59 ± 0.29
								
**CO2**		33.65 ± 9.61	201.11 ± 24.33	2.07 ± 0.08	415.94 ± 168.73	1.78 ± 0.28	741.64 ± 345.31	3.92 ± 0.59	1.57 ± 1.08
**MO2**		31.29 ± 10.30	357.08 * ± 109.87	2.56 * ± 0.26	923.12 * ± 328.64	2.42 * ± 0.35	2311.24 * ± 1114.42	5.79 * ± 1.37	1.86 ± 0.98
								
**CO3**		22.17 ± 2.65	131.45 ± 85.35	2.12 ± 0.11	274.68 ± 179.52	1.95 ± 0.16	519.93 ± 330.60	1.77 ± 0.98	0.69 ± 0.39
**MO3**		21.97 ± 7.06	210.92 * ± 75.54	2.35 * ± 0.19	493.44 * ± 170.69	2.01 ± 0.21	992.77 * ± 346.29	1.37 ± 0.69	0.59 ± 0.29
								
**CO4**		17.85 ± 6.97	140.28 ± 35.32	3.01 ± 0.32	413.00 ± 81.42	2.39 ± 0.40	1000.62 ± 317.84	1.67 ± 1.18	0.59 ± 0.49
**MO4**		10.20 * ± 3.43	79.46 * ± 48.07	2.95 ± 0.24	229.55 * ± 136.36	2.49 ± 0.30	555.25 * ± 334.52	1.67 ± 1.18	0.69 ± 0.49
								
**CO5**		44.83 ± 17.17	252.12 ± 169.71	3.33 ± 0.22	844.64 ± 566.04	2.59 ± 0.22	2146.43 ± 1428.34	5.00 ± 1.18	1.67 ± 0.69
**MO5**		29.04^*^ ± 11.18	264.87 ± 51.01	3.55 ± 0.35	952.55 ± 258.00	3.00 * ± 0.67	2994.99 * ± 1451.88	5.79 ± 1.20	2.35 * ± 0.78
**^1^ MIL**		10.69 ^b^ ± 3.63	121.64 ^b^ ± 12.75	1.95 ^b^ ± 0.40	236.42 ^b^ ± 56.90	1.83 ^b^ ± 0.15	432.62 ^b^ ± 109.87	1.86 ± 1.28	0.78 ^a^ ± 0.49
**^1^ FEL**		10.59 ^b^ ± 3.43	45.13 ^c^ ± 36.30	4.06 ^a^ ± 1.17	223.67 ^b^ ± 204.05	2.37 ^a^ ± 0.31	571.92 ^b^ ± 125.82	1.37 ± 0.29	0.59 ±0.10
^1^ **CON**		19.13 ^a^ ± 3.63	202.09 ^a^ ± 51.99	2.50 ^b^ ± 0.30	503.25 ^a^ ± 127.53	1.89 ^b^ ± 0.17	962.36 ^a^ ± 314.90	1.47 ± 1.47	0.39 ^b^ ± 0.29
*p-value*		*<0.001*	*<0.001*	*<0.001*	*<0.001*	*<0.001*	*<0.001*	ns	*<0.001*

For Mora Romagnola (MO1–MO5), means followed by * significantly differ from their conventional counterparts (CO1–CO5) (*p* < 0.05). ^1^ For market leaders salami, means followed by different letters significantly differ among them (Tukey-HSD, *p* < 0.05). ns: “not significant”.

## Data Availability

Data is contained within the article.

## References

[1] Pieniak Z., Verbeke W., Vanhonacker F., Guerrero L., Hersleth M. (2009). Association between traditional food consumption and motives for food choice in six European countries. Appetite.

[2] Almli V.L., Verbeke W., Vanhonacker F., Næs T., Hersleth M. (2011). General image and attribute perceptions of traditional food in six European countries. Food Qual. Prefer..

[3] Chambers S., Lobb A., Butler L., Harvey K., Traill B. (2007). Local, national and imported foods: A qualitative study. Appetite.

[4] Guerrero L., Guàrdia M.D., Xicola J., Verbeke W., Vanhonacker F., Zakowska-Biemans S., Scalvedi M.L. (2009). Consumer-driven definition of traditional food products and innovation in traditional foods. A qualitative cross-cultural study. Appetite.

[5] Di Monaco R., Cavella S. (2015). Differences in liking of traditional salami: The effect of local consumer familiarity and relation with the manufacturing process. Br. Food J..

[6] Monteleone E., Dinnella C., Meiselman H.L. (2009). Italian meals. Meals in Science and Practice, Interdisciplinary Research and Business Applications.

[7] Conter M., Zanardi E., Ghidini S., Pennisi L., Vergara A., Campanini G., Ianieri A. (2008). Consumers’ behaviour toward typical Italian dry sausages. Food Contr..

[8] Sajali N., Wong S.C., Abu Bakar S., Khairil Mokhtar N.F., Manaf Y.N., Yuswan M.H., Mohd Desa M.N. (2020). Analytical approaches of meat authentication in food. Int. J. Food Sci. Technol..

[9] Gama L.T., Martinez A., Carolino M.I., Periati V.L., Bermejo J.V.D., Vicente A.P., Vega-Pla J., Cortés O., Sousa C.O. (2013). Genetic structure, relationships and admixture with wild relatives in native pig breeds from Iberia and its islands. Genet. Sel. Evol..

[10] Franci O., Pugliese C. (2007). Italian autochthonous pigs: Progress report and research perspectives. Ital. J. Anim. Sci..

[11] Prosperi P., Allen T., Padilla M., Peri I., Cogill B. (2014). Sustainability and food & nutrition security: A vulnerability assessment framework for the Mediterranean region. Sage Open.

[12] Olivares A., Daza A., Rey A.I., Lopez-Bote C.J. (2009). Interactions between genotype, dietary fat saturation and vitamin A concentration on intramuscular fat content and fatty acid composition in pigs. Meat Sci..

[13] Poklukar K., Čandek-Potokar M., Batorek-Lukač N., Tomažin U., Škrlep M. (2020). Lipid deposition and metabolism in local and modern pig breeds: A review. Animals.

[14] Muñoz M., Bozzi R., García-Casco J., Núñez Y., Ribani A., Franci O., García F., Škrlep M., Schiavo G., Bovo S. (2019). Genomic diversity, linkage disequilibrium and selection signatures in European local pig breeds assessed with a high density SNP chip. Sci. Rep..

[15] Tinarelli S., Ribani A., Utzeri V.J., Taurisano V., Bovo C., Dall’Olio S., Nen F., Bovo S., Schiavo G., Gallo M. (2021). Redefinition of the Mora Romagnola Pig Breed Herd Book Standard Based on DNA Markers Useful to Authenticate Its “Mono-Breed” Products: An Example of Sustainable Conservation of a Livestock Genetic Resource. Animals.

[16] Pugliese C., Sirtori F. (2012). Quality of meat and meat products produced from southern European pig breeds. Meat Sci..

[17] Chen Q., Hu Y., Wen R., Wang Y., Qin L., Kong B. (2021). Characterisation of the flavour profile of dry fermented sausages with different NaCl substitutes using HS-SPME-GC-MS combined with electronic nose and electronic tongue. Meat Sci..

[18] Barbieri S., Bendini A., Balestra F., Palagano R., Rocculi P., Toschi T.G. (2018). Sensory and instrumental study of Taralli, a typical Italian bakery product. Eur. Food Res. Technol..

[19] (2010). Disciplinare di Produzione della Indicazione Geografica Protetta “Salame Felino”.

[20] (1996). Definisce la Composizione e le Caratteristiche del Salame Milano.

[21] Prakash V., Martín-Belloso O., Keener L., Astley S.B., Braun S., McMahon H., Lelieveld H. (2015). Regulating Safety of Traditional and Ethnic Foods.

[22] Del Nobile M.A., Conte A., Incoronato A.L., Panza O., Sevi A., Marino R. (2009). New strategies for reducing the pork back-fat content in typical Italian salami. Meat Sci..

[23] De Campos R.M.L., Terra N.N., Campagnol P.C.B. (2008). Sensory Aspects of Cooked Meats. Handbook of Muscle Foods Analysis, Nollet, L.M.L., Toldrà F., Eds..

[24] Herrero A.M., De la Hoz L., Ordóñez J.A., Herranz B., de Ávila M.R., Cambero M.I. (2008). Tensile properties of cooked meat sausages and their correlation with texture profile analysis (TPA) parameters and physico-chemical characteristics. Meat Sci..

[25] Moretti V.M., Madonia G., Diaferia C., Mentasti T., Paleari M.A., Panseri S., Pirone G., Gandini G. (2004). Chemical and microbiological parameters and sensory attributes of a typical Sicilian salami ripened in different conditions. Meat Sci..

[26] Lorenzo J.M., Bedia M., Bañón S. (2013). Relationship between flavour deterioration and the volatile compound profile of semi-ripened sausage. Meat Sci..

[27] Ordóñez J.A., Hierro E.M., Bruna J.M., Hoz L.D.L. (1999). Changes in the components of dry-fermented sausages during ripening. Crit. Rev. Food Sci. Nutr..

[28] Gøtterup J., Olsen K., Knøchel S., Tjener K., Stahnke L.H., Møller J.K. (2008). Color formation in fermented sausages by meat-associated staphylococci with different nitrite-and nitrate-reductase activities. Meat Sci..

[29] Jerković I., Kovačević D., Šubarić D., Marijanović Z., Mastanjević K., Suman K. (2010). Authentication study of volatile flavour compounds composition in Slavonian traditional dry fermented salami “kulen”. Food Chem..

[30] Olivares A., Navarro J.L., Flores M. (2009). Establishment of the contribution of volatile compounds to the aroma of fermented sausages at different stages of processing and storage. Food Chem..

[31] Marco A., Navarro J.L., Flores M. (2008). The sensory quality of dry fermented sausages as affected by fermentation stage and curing agents. Eur. Food Res. Technol..

[32] Procida G., Conte L.S., Fiorasi S., Comi G., Favretto L.G. (1999). Study on volatile components in salami by reverse carrier gas headspace gas chromatography–mass spectrometry. J. Chromatogr. A.

[33] Montel M.C., Masson F., Talon R. (1998). Bacterial role in flavour development. Meat Sci..

[34] Frankel E.N. (2012). Lipid Oxidation.

[35] Meynier A., Novelli E., Chizzolini R., Zanardi E., Gandemer G. (1999). Volatile compounds of commercial Milano salami. Meat Sci..

[36] Stahnke L.H. (1995). Dried sausages fermented with Staphylococcus xylosus at different temperatures and with different ingredient levels-Part II. Volatile components. Meat Sci..

[37] Hofmann T., Schieberle P. (2000). Formation of aroma-active Strecker-aldehydes by a direct oxidative degradation of Amadori compounds. J. Agric. Food Chem..

[38] Lanzotti V. (2006). The Analysis of Onion and Garlic. J. Chrom. A..

[39] Simonsen J.L., Ross W.C.J. (1957). The Terpenes.

[40] Tholl D., Boland W., Hansel A., Loreto F., Röse U.S., Schnitzler J.P. (2006). Practical approaches to plant volatile analysis. Plant J..

[41] El-Zaeddi H., Martínez-Tomé J., Calín-Sánchez Á., Burló F., Carbonell-Barrachina Á.A. (2017). Irrigation dose and plant density affect the volatile composition and sensory quality of dill (*Anethum graveolens* L.). J. Sci. Food Agric..

[42] Kim J., Knowles S., Ahmad R., Day L. (2021). Objective Measurements Associated with the Preferred Eating Qualities of Fermented Salamis. Foods.

[43] Saccani G., Fornelli G., Zanardi E. (2013). Characterization of textural properties and changes of myofibrillar and sarcoplasmic proteins in salame felino during ripening. Int. J. Food Prop..

[44] González-Fernández C., Santos E.M., Rovira J., Jaime I. (2006). The effect of sugar concentration and starter culture on instrumental and sensory textural properties of chorizo-Spanish dry-cured sausage. Meat Sci..

[45] Aro J.M.A., Nyam-Osor P., Tsuji K., Shimada K.I., Fukushima M., Sekikawa M. (2010). The effect of starter cultures on proteolytic changes and amino acid content in fermented sausages. Food Chem..

[46] Montanari C., Gatto V., Torriani S., Barbieri F., Bargossi E., Lanciotti R., Grazie L., Magnani R., Tabanelli G., Gardini F. (2018). Effects of the diameter on physico-chemical, microbiological and volatile profile in dry fermented sausages produced with two different starter cultures. Food Biosci..

[47] Pasini F., Soglia F., Petracci M., Caboni M.F., Marziali S., Montanari C., Gardini F., Grazia L., Tabanelli G. (2018). Effect of fermentation with different lactic acid bacteria starter cultures on biogenic amine content and ripening patterns in dry fermented sausages. Nutrients.

[48] Hughes M.C., Kerry J.P., Arendt E.K., Kenneally P.M., McSweeney P.L.H., O’neill E.E. (2002). Characterization of proteolysis during the ripening of semi-dry fermented sausages. Meat Sci..

[49] Cocolin L., Rantsiou K., Hui Y.H. (2012). Meat Fermentation. Handbook of Meat and Meat Processing.

[50] Lorenzo J.M., Temperán S., Bermúdez R., Cobas N., Purriños L. (2012). Changes in physico-chemical, microbiological, textural and sensory attributes during ripening of dry-cured foal salchichón. Meat Sci..

